# 

**Published:** 1877-04-20

**Authors:** 


					﻿VOL. VH.	APRIL 20, 1877.	No. 4.
THE
SOUTHERN MEDICAL RECORD.
A MONTHLY JOURNAL OF PRACTICAL MEDICINE.
OUJ C217.LD PR^CIPIES ESTO BE&VIS.”
a
a
a
^a
Qu
fA
w
bH
<
■O
A*
A
a
o
><
o
H
a
■ a
a
o
*-»
02
I—(
a
£■*
a
o
02
<
a
o
a
a
£
a
02
a
a
a
a
02
a
■a
a
02
a
o
a
a
n
o
8
2
d
3
i—<
a
►
a
o
02
>
a
d
a
a
►
o
a
hi
a
>
d
HI
a
w
S
02
00
O
d
£
HI
a
d
o
JAS. P. HARRISON & CO., Printers.
ATLANTA, GEORGIA.
TERMS: $2.00 per Annum in Advance—Club Rates, 5 Copies for $8.00.
				

